# Exposure-response relation for vibration-induced white finger: influence of different estimates of daily exposure time

**DOI:** 10.1007/s00420-026-02210-w

**Published:** 2026-05-02

**Authors:** Magdalena F. Scholz, Anthony J. Brammer, Steffen Marburg

**Affiliations:** 1 School of Engineering and Design, Technical University of Munich, Boltzmannstraße 15, Garching, 85748 Bavaria Germany; 2 Department of Medicine, University of Connecticut Health, 263 Farmington Avenue, Farmington, 06030 Connecticut USA

**Keywords:** Hand-arm vibration, Vibration white finger, Exposure-response relation, Prevalence

## Abstract

**Purpose:**

It is analyzed whether differences in how the daily usage time is determined in a population exposed to hand-transmitted vibration are the cause of discrepancies between exposure-response models constructed for vibration-induced white finger (VWF). The predictions of the models are then used to evaluate the one in ISO 5349-1:2001.

**Methods:**

Pooled analyses employing data from epidemiologic studies of vibration-exposed occupations deemed the most reliable, together with published ratios between daily tool or machine usage times determined by questionnaire or interview and measured values, are used to construct exposure-response relations. These models are compared to those in which the daily usage time is not compensated.

**Results:**

Of 17 data points, for 7 the usage time ratio is greater than 1, and for the rest the ratio is equal to 1. Adjusting daily vibration exposures, expressed as A(8)-values, by means of such ratios, results in reductions of varying magnitude. However, the exposure-response models show minor changes, and the scatter in the data points previously observed remains.

**Conclusions:**

Methods for estimating 10% prevalence of VWF from the prevalence recorded in a population study are found to have more effect on the models than different methods for estimating the daily usage time. Yet, the spread and clustering in the data remain and point to issues in the constituents of the *A*(8)-value. The ISO relation is generally consistent with the lower 95-percentile confidence limit of the models and so provides a conservative exposure limit at least for *A*(8)-values greater than about 3 $\frac{m}{s^2}$.

## Introduction

Occupational exposure of the hand or hands to vibration is common in industry and wherever hand-held power tools and machines are used. With exposure continuing for a number of years often leading to the development of the hand-arm vibration syndrome (HAVS), there has long been interest in specifying tolerable daily vibration exposure limits. In 2001, an exposure-response relation was proposed by the International Organization for Standardization (ISO) (ISO 5349-1:2001), which has served as the basis for most occupational exposure guidelines and regulations in effect today. While the relation was from a population-based model built on the epidemiologic data available at the time (Brammer (1982a, 1982b)), there has been ongoing debate concerning its accuracy and applicability to different occupations. This has continued as the results of more epidemiologic studies have become available (Bovenzi et al. (1988, 1995); Bovenzi (1998b, 2012); Engström and Dandanell (1986); Futatsuka et al. (1984); Gemne and Lundström (1996); Gerhardsson et al. (2020); Starck et al. (1990); Tominaga (1990); Walker et al. (1985)). In consequence, there have been numerous attempts to relate occupational exposure of the hands to common signs and symptoms of HAVS, including the finger blanching associated with vibration-induced white finger (VWF) (Bovenzi (1994); Bovenzi et al. (1995); Bovenzi (1998a, 2010b); Brammer (1982a, 1982b, 1986); Futatsuka et al. (1984); Griffin (1982); Griffin et al. (2003); Miyashita et al. (1982); Nilsson et al. (2017); Sauni et al. (2009); Scholz et al. (2023a, 2023b); Su et al. (2013); Taylor et al. (1975); Tominaga (1982)). Most focus on the exposure of a single occupation and so cannot be generalized to exposures involving different power tools and machines. Nilsson et al. (2017), Scholz et al. (2023a, 2023b), Brammer (1982a, 1982b), Griffin et al. (2003) and Taylor et al. (1975) do derive relations from exposures to different vibration sources. Nilsson et al. (2017) and Scholz et al. (2023a, 2023b) provide relations that can be compared to the ISO exposure-response relation and are discussed in this report. The last two provide no means to compare with the ISO predictions.

In a recent series of papers, Scholz and co-workers have been conducting a comprehensive evaluation of the factors influencing population-based models for the onset of VWF using data derived from epidemiologic studies. Their predictions are compared with those of the ISO model (Scholz et al. (2023a, 2023b, 2024)). This contribution is a continuation of their work. The starting point of these investigations has been to accept the findings of a comprehensive and authoritative meta-analysis of epidemiologic studies of VWF published between 1945 and 2016 by Nilsson et al. (2017). In this way, any bias of the present authors in the selection of epidemiologic studies to consider for inclusion in models is avoided. Scholz and co-workers then apply their own selection rules to the studies identified by Nilsson et al. (2017) as possessing “high quality”. The rules are structured to confirm that the data were acquired in a manner consistent with the methods contained in ISO 5349-1:2001 and that the finger blanching was associated with vibration exposure. Hence, direct comparison of the results of their exposure-response models with the ISO model can be obtained. The latter predicts the mean duration of employment in years for 10% of a population to develop VWF. The models consider each member of a population to be performing essentially the same work (e.g., forestry chain saw operators; miners using rock drills; etc.) and so, at the population level, can be assumed to be regularly exposed to a given daily vibration exposure. This is constructed from the tool(s) or machine(s) vibration magnitude(s) and daily exposure time(s). The latter metric for the population is typically obtained by questionnaires or interviews, or by observations conducted by a third party on a subset of the population. It is termed here the daily usage time, in order to differentiate between the time a tool is in the operators’ hands, which is commonly reported, and the physically measurable tool or machine operating time. All variations in individual exposures and responses are thus subsumed by other model parameters.

None of the published epidemiologic studies identified by Nilsson et al. (2017) were performed when the population prevalence of VWF was 10%. Hence, it is necessary to transform the mean duration of employment at the time of the study to that which would have occurred when the population prevalence was 10%, in order to construct exposure-response models directly comparable with the ISO model. The procedures used to select data within studies, together with details of the data transformation and initial models, as well as comparisons with the ISO exposure-response model, are described in Scholz et al. (2023a, 2023b).

The scatter of the data about the mean values predicted by the initial models, termed here the variability, was attributed to several problems of the data and limitations of the modeling that would require further study. While many potential sources of inconsistency have been reported (Scholz et al. (2023a)), the contribution of each to an exposure-response model for VWF has neither been previously demonstrated nor quantified. The first, and believed to be the most significant, concerned the method for transforming the mean duration of employment at the time of the epidemiologic study to that when the prevalence of VWF would have been 10%. Scholz and co-workers’ initial pooled analyses employed linear interpolation from the reported mean duration of employment in years and prevalence of VWF to the value for 10% of the population to experience VWF. Linear interpolation was acknowledged to be an oversimplification, with the growth of VWF in a population expected to follow an “S”-shaped curve as the duration of employment increased. The definition of this curve was undertaken by constructing a polynomial function that produces an “S”-shape for the prevalence when specified in terms of the duration of employment in years (Scholz et al. (2024)). This function has enabled polynomial interpolation to 10% prevalence of VWF.

The scatter of the data from different populations was not reduced in the pooled analyses when polynomial prevalence interpolation was substituted for linear prevalence interpolation to estimate the mean duration of employment to reach 10% VWF. This suggests that the source, or sources of inconsistency, lie(s) elsewhere (Scholz et al. (2023b)). In consequence, it appears appropriate now to focus attention on the parameters employed in the ISO procedure to define the daily exposure, namely the tool or machine vibration magnitude(s) and the daily usage time(s). Of these two parameters, the daily usage time is the subject of the present investigation, as differences as large as a factor of 2.5 have been observed between different methods for establishing its magnitude (McCallig et al. (2010); Palmer et al. (2000)).

While a single epidemiologic study could be subject to an error in the daily exposure of up to 60%, since the ISO construct employs the square root of the usage time, it is not evident what the effect will be on the models as not all studies are subject to an error of this magnitude. Many included in the pooled analyses employed observer’s measurements of daily usage times, which are taken to be the “gold standard”, though these measurements could not have been performed throughout exposures lasting several years. With this limitation, the effects of adjustments to the daily usage time on the performance of the models are explored in the present work.

In this contribution, the method for adjusting the daily usage time is first described together with the other constituents of the modeling. Results are presented for models employing data with different pre-processing that might be considered to influence their reliability. Comparisons are provided between models constructed with and without daily usage time adjustments, so that the magnitude of the effect on each model can be assessed. Comparisons with the predictions of the ISO and Nilsson et al. (2017) models, as well as those of the present authors, are also presented. Additionally, a rationale is developed for interpreting the performance of the ISO model from the confidence intervals produced by the models described here (Scholz et al. (2023b)).

## Methods

### Dataset for pooled analysis

As described in Scholz et al. (2023a) and Scholz et al. (2023b), pooled analyses of populations exposed to hand-transmitted vibration have been performed. They employed the dataset judged to possess sufficient “quality” in the comprehensive meta-analysis conducted by Nilsson et al. (2017), who evaluated all population studies published in English since the Second World War. In order to ensure reliability of the data, and compatibility and comparability with the ISO methodology, further selection rules were introduced. In summary, the first five of these eleven rules state that the measurement of vibration and the calculation of the daily exposure have to be in accordance with the procedures defined in ISO 5349-1:2001. Rules six through eleven deal with the epidemiologic data. They state that the population size has to be a minimum of thirty full-time employees performing similar work involving near-daily exposure to hand-transmitted vibration. Furthermore, a differential diagnosis has to have been performed to distinguish VWF from Raynaud’s phenomenon due to other causes. Otherwise, a control group that allows for compensation for symptoms unlikely to be associated with the vibration exposure has to have been part of the study. Of the analyzed population, a minimum of 10% of its members need to have been affected by VWF. A lower percentage would require extrapolation to create an exposure-response model for 10% prevalence, which would increase the potential for error. For this reason, such studies are excluded. Lastly, the mean group exposure time to reach the prevalence of VWF recorded in a population is to have been reported, preferably in years.

The studies included in the models described here complied with the selection rules established by both Nilsson et al. (2017) and Scholz et al. (2023a). Therefore, they are deemed to provide reliable data with which to create exposure-response relations. Nonetheless, there are differences in applied methodologies within those studies that are described below.

### Evaluation of daily vibration exposure

Among the differences between studies is how the daily usage time of power tool(s) and/or machine(s) was determined.

The daily usage time, *T*, influences exposure-response relations as it is incorporated with the 8-h, frequency-weighted, energy-equivalent acceleration sum into the calculation of the daily exposure, as defined in ISO standard ISO 5349-1 (2001), and referred to as the *A*(8)-value:

1 $$\begin{aligned} A(8) = a_{hv} \cdot \sqrt{\frac{T}{T_{0}}} = \sqrt{\frac{1}{T_{0}} \sum _{i = 1}^{n} a_{hvi}^2 \cdot T_i} \end{aligned}$$

In this equation for the daily vibration exposure, *A*(8), the daily usage time, *T*, is expressed relative to an 8h workday ($T_0$) and combined with the triaxial and frequency-weighted vibration total value, $a_{hv}$. If *n* multiple tools or machines are used per day, their respective daily usage times and vibration values are indicated by a subscript *i*, multiplied and summed as shown in Eq. 1.

Here, for each of the studies from which data are included, the method or methods used to determine the daily usage time are noted, as well as the tools or machines used by the respective population during a typical workday. Both McCallig et al. (2010) and Palmer et al. (2000) found questionnaires to be less accurate than interviews, which in turn were less accurate than observation or measurement. In this work, the most accurate method employed in the respective study is used in the following, which is either interview or observation for this set of studies. For those tools or machines for which only interview data are available, the ratio between the daily usage time determined by an interview and that measured or observed is estimated from either McCallig et al. (2010) or Palmer et al. (2000). This results in a

2 $$\begin{aligned} T-ratio = \frac{T_{\mathrm{interview}}}{T_{\mathrm{measured/observed}}} \end{aligned}$$

which is then used to adjust the *A*(8)-value reported in each study by multiplying it with the square root of the inverse of that ratio, as shown in the following equation:

3 $$\begin{aligned} A(8)_{adj} = A(8) \cdot \sqrt{\frac{1}{T-ratio}} \end{aligned}$$

in which the $T-ratio$ is either the individual ratio, if only one tool or machine was used during a workday, or an average of the ratios of all the tools or machines used by the respective population.

### Prevalence growth in a vibration-exposed population

An exposure-response model is desired to predict the duration of employment exposed to vibration for a given prevalence of VWF to occur in a vibration-exposed population. A VWF prevalence of 10% is chosen here to enable direct comparison with the model in ISO 5349-1:2001 and our previous work. Now, the prevalence of VWF recorded in the populations for which acceptable data are available was not the desired value (10%). Hence, the mean exposure time in years recorded for each population needs to be transformed to that at which the prevalence would have been 10%. Two estimates for prevalence growth with time in a vibration-exposed population have been constructed. Both assume that the prevalence of VWF at the commencement of vibration exposure is zero (as is ensured by the selection rules). The first employed linear interpolation from zero prevalence and exposure time to the recorded VWF prevalence and duration of employment, from which the exposure time corresponding to the desired prevalence for modeling can be directly obtained. The second involved constructing an invertible polynomial for the prevalence-exposure-time function of a given population, as described in Scholz et al. (2024). The curvilinear prevalence growth function is adapted for each population by introducing $A(8)_{adj}$ into the following expression for the prevalence, *P*:

4 $$\begin{aligned} & P =(\frac{A(8)_{adj}}{3.59})^{0.3}\cdot ((0.02985+a_1') \cdot D_y + 0.1001\cdot D_y^2 \nonumber \\ & + (-0.003213) \cdot D_y^3 + 0.00002886 \cdot D_y^4) \end{aligned}$$

In this equation, $D_y$ is the mean group exposure time in years, and $a_1'$ is a fitted non-dimensional parameter that accounts for study-specific differences in exposure (e.g., ergonomic, biodynamic and environmental factors) and changes in population membership with time. Establishing the value of $a_1'$ for a given population, with the value of $A(8)_{adj}$ derived from Eq. 3, enables the expression for *P* to be inverted and a second estimate of the group exposure time, $D_{y,adj}$, corresponding to the desired prevalence of VWF to be obtained.

### Exposure-response models for the development of VWF

The *A*(8)- and $D_y$-value adjustments applied to the data for each population are then employed in a regression analysis to construct an exposure-response model for a given prevalence of VWF, as in Scholz et al. (2023a) and Scholz et al. (2023b). For comparison with the model contained in ISO 5349-1:2001 and with previous work for estimating the mean exposure time to reach 10% prevalence of VWF in a population, the expression is:

5 $$\begin{aligned} D_{y,10,j,k} = a \cdot A(8)_{k}^b \end{aligned}$$

in which $D_{y,10,j,k}$ is the mean group exposure time in years at which it is predicted that a 10% prevalence of VWF will occur in a population with a daily exposure $A(8)_{k}$, and *a* and *b* are numerical fit parameters. The subscripts indicate the percentage to which the mean group exposure time was interpolated (10%), the interpolation method used (subscript j, linear or polynomial), and whether the daily usage time was adjusted (subscript k). The mean exposure times are estimated by linear and polynomial prevalence interpolation, denoted by $D_{y,10,lin}$ and $D_{y,10,poly}$, respectively for unadjusted *A*(8)-values, and by $D_{y,10,lin}$ and $D_{y,10,poly,adj}$ for the adjusted *A*(8)-values. The subscript k stays the same in the case of linear interpolation, as the adjustment of the *A*(8)-value does not influence this interpolation.

Values for $D_{y,10,j}$ without adjustment for the daily usage times have already been calculated for linear prevalence interpolation in Scholz et al. (2023a) and for polynomial interpolation in Scholz et al. (2023b), and are used for the present analyses. For those data subsets that encompass enough data points with a sufficient range of values and a coefficient of determination for the exposure-response model ($r^2$) in excess of 0.49 (i.e., a correlation coefficient > 0.7), 95-percentile confidence intervals are calculated.

As in previous publications by the authors, the model created from the full dataset is compared against the uninterpolated data for the prevalence of VWF and mean time exposed to vibration reported by each population. An exposure-response model predicting the mean population exposure times at different *A*(8)-values for 10% prevalence of VWF to occur that falls below all reported data points with prevalences of 10%, and more, may be considered to provide a conservative estimate of the risk posed by exposure to hand-transmitted vibration. The models are further compared to the ISO model and to that created by Nilsson et al. (2017).

## Results

### Full dataset and daily usage time adjustment

The studies that fulfilled both the criteria established in the meta-analysis by Nilsson et al. (2017) and those first introduced in the pooled analyses by Scholz et al. (2023a) are listed in Table 1. For every population in the respective study, the reported mean exposure time, $D_y$ in years, is included. For Bovenzi (1998b), the values are calculated from the total hours of tool usage provided in the publication, as described in Scholz et al. (2023a). Furthermore, Table 1 contains the respective populations sizes, the reported prevalences and the interpolated mean population exposure times estimated for 10% prevalence of VWF for both linear and polynomial prevalence interpolation, $D_{y,10,lin}$ and $D_{y,10,poly}$, from Scholz et al. (2023a) and Scholz et al. (2023b), respectively. The table also includes $D_{y,10,poly,adj}$, the mean population exposure times estimated for 10% prevalence of VWF by polynomial interpolation for studies in which the daily exposure has been adjusted to account for the method for determining the daily usage time. This is done by inverting Eq. 4 using the values for $A(8)_{adj}$ in Table 3.

**Table 1:** Studies, reported mean population exposure times $D_y$, population sizes and point prevalences derived from the publications, and estimated values for $D_{y,10,lin}$ from Scholz et al. (2023a), $D_{y,10,poly}$ from Scholz et al. (2023b) and $D_{y,10,poly,adj}$

Study	$D_{y}$ / years	Population Size	Prevalence / %	$D_{y,10,lin}$ / years	$D_{y,10,poly}$ / years	$D_{y,10,poly,adj}$ / years
Bovenzi (1994)	17.4	570	30.2	5.9	7.6	7.4
Bovenzi (1994)	18.3	145	40.7	4.5	6.2	6.0
Bovenzi (1994)	14.9	188	13.8	10.8	11.8	11.8
Bovenzi (1994)	18.9	237	36.7	5.2	7.1	6.9
Bovenzi et al. (1995)	11.1	222	23.4	4.7	5.6	5.6
Bovenzi (1998b)	17.9	132	12.1	14.8	15.5	15.5
Bovenzi (1998b)	17.8	65	23.1	7.7	9.5	9.5
Bovenzi (1998b)	21.5	140	15.0	14.3	15.5	15.5
Bovenzi (1998b)	24.6	41	36.6	6.7	9.0	9.0
Bovenzi (1998b)	15.0	31	51.6	2.9	3.5	3.5
Bovenzi (1998b)	9.1	165	23.0	4.0	4.5	4.5
Bovenzi (2008)	10.9	128	26.6	4.1	4.8	4.8
Bovenzi et al. (2008)	16.0	216	18.1	8.8	10.5	10.5
Bovenzi et al. (2008)	15.8	183	14.8	10.7	12.0	12.0
Bovenzi et al. (2008)	17.5	33	36.4	4.8	6.4	6.4
Bovenzi (2010a)	15.0	249	17.3	8.7	10.1	10.1
Chatterjee et al. (1978)	7.5^a^	42	50.0	1.5^b^	2.4^c^	2.3$^c$

All data for male workers

^a^ Median group exposure time

^b^ Calculated with median group exposure time

^c^Calculated with mean group exposure time of 9.9 years

In order to obtain these results, the tools used in each of the studies have been identified, and the corresponding ratio between a daily usage time reported in an interview versus a measured time has been sourced from Palmer et al. (2000) and McCallig et al. (2010). These are listed in Table 2. As McCallig et al. (2010) provide a value for two angle grinders of different sizes (9-inch and 5-inch diameter grinding wheels), and none of the studies includes any information on the size of the angle grinder used, the two values, 1.4 and 1.5, are averaged for the present work. For all tools for which no individual value is reported in either of the two sources, the general value for any hand-held tool in Palmer et al. (2000) is applied (2.5). A rock drill is considered to be different from a hammer drill, for which a $T-ratio$ is reported in Palmer et al. (2000), and also from both a battery and an electric drill that are included in the list of ratios in McCallig et al. (2010). For this tool, the general value is applied here as well.

**Table 2:** Tool, ratio between daily usage time *T* reported in an interview and an observed or measured usage time ($T-ratio$ according to Eq. 2), and source of that ratio

Tool	$T-ratio$	Source
Rock breaker	2.5^a^	Palmer et al. (2000)
Rock drill	2.5^a^	Palmer et al. (2000)
Angle grinder	1.45	McCallig et al. (2010)
(Light) Stone hammer	2.5^a^	Palmer et al. (2000)
Chain saw / AV-chain saw	1.1	McCallig et al. (2010)
Percussive drill	2.5^a^	Palmer et al. (2000)

^a^ Value for any hand-held tool

Table 3 contains for each study and population the reported *A*(8)-value, the tools, and the method used to determine the daily usage time, *T*. The fifth column of that table contains the list of $T-ratio$s for the respective tool(s). The final two columns contain the averaged $T-ratio$ and the adjusted *A*(8)-value calculated using Eq. 3, $A(8)_{adj}$. As already noted, the latter forms one of the independent variables in the exposure-response predictive models (Eq. 5). It is also used in Eq. 4 to estimate the mean exposure time for 10% prevalence of VWF to occur in a population by polynomial interpolation ($D_{y,10,poly,adj}$ in Table 1).

**Table 3:** Studies, *A*(8)-values, tools used, the method for determining the daily usage time *T* (I = interview, R = employment records, Q = questionnaire, M = measurement/observation), the individual and the average$T-ratio$s between the methods used to determine the daily usage time and a measured daily usage time for all tools, and an adjusted *A*(8),$A(8)_{adj}$

Study	*A*(8) /$\frac{m}{s^2}$	Tools used	*T*-method	Indiv.$T-ratio$s	Mean$T-ratio$s	A(8)_adj_ /$\frac{m}{s^2}$
Bovenzi (1994)	8.4	Rock breakers, rock drills, angle grinders, light stone hammers	I,R	2.5, 2.5, 1.45, 2.5	2.2	5.6
Bovenzi (1994)	12.4	Rock breakers, rock drills	I,R	2.5, 2.5	2.5	7.8
Bovenzi (1994)	2.1	Angle grinders	I,R	1.45	1.5	1.8
Bovenzi (1994)	10.8	Angle grinders, light stone hammers	I,R	1.45, 2.5	2.0	7.6
Bovenzi et al. (1995)	4.4	Chain saws, AV-chain saws	Q, I, R, fuel	1.1	1.1	4.2
Bovenzi (1998b)	1.9	Selection from: caulking tools, chipping hammers, impact wrenches, nut runners, scaling hammers, hand-held grinders and polishers	Q, I, R, M	–	1	1.9
Bovenzi (1998b)	4.2		Q, I, R, M	–	1	4.2
Bovenzi (1998b)	1.7		Q, I, R, M	–	1	1.7
Bovenzi (1998b)	8.3	Selection from: rock drills, road breakers, hammer drills, stone hammers, hand-held grinders and polishers	Q, I, R, M	–	1	8.3
Bovenzi (1998b)	4.7		Q, I, R, M	–	1	4.7
Bovenzi (1998b)	4.1	Chain saws, brush saws	Q, I, R, M		1	4.1
Bovenzi (2008)	3.7	Chain saws, AV-chain saws	Q, I, R	1.1	1.1	3.5
Bovenzi et al. (2008)	4.4	Brush saws, chain saws, grinders, polishers, inline hammers	Q, I, R, M	–	1	4.4
Bovenzi et al. (2008)	3.6	Brush saws, chain saws	Q, I, R, M	–	1	3.6
Bovenzi et al. (2008)	8.8	Grinders, polishers, inline hammers	Q, I, R, M	–	1	8.8
Bovenzi (2010a)	3.8	Brush saws, chain saws, grinders, polishers, inline hammers	Q, I, R, M	–	1	3.8
Chatterjee et al. (1978)	18.7	Percussive drills	Q, I	2.5	2.5	11.8

All data for male workers

### Data subsets

**Fig. 1:**
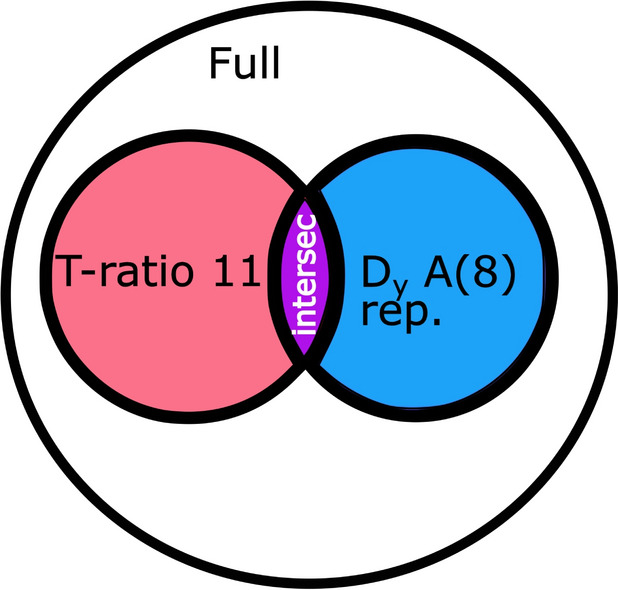
The data subsets used for analyses in past and present work are displayed schematically. The full dataset (Bovenzi (1994); Bovenzi et al. (1995); Bovenzi (1998b, 2008); Bovenzi et al. (2008); Bovenzi (2010a); Chatterjee et al. (1978)) includes, among other studies, those in which both the $D_y$ and the *A*(8)-values are determined and reported in accordance with the selection rules (Bovenzi (1994); Bovenzi et al. (1995); Bovenzi (2008); Bovenzi et al. (2008); Bovenzi (2010a); Chatterjee et al. (1978)). These form the data subset $D_y$ *A*(8) rep., which is shown as a circle with a blue filling color. Another subset of studies in the full dataset is the one in which the $T-ratio$ is 1 or 1.1. This subset is called $T-ratio$ 11, as the highest $T-ratio$ is 1.1, and is shown as a circle filled in a red color. The studies with common properties in these subsets are illustrated by an area in which they intersect. It is given a violet color and labeled intersec (Bovenzi et al. (1995); Bovenzi (2008); Bovenzi et al. (2008); Bovenzi (2010a)). This area defines a data subset in which studies reported and determined $D_y$ in years, *A*(8) as required by the selection rules, and also a $T-ratio$ that is no greater than 1.1. It comprises the studies by Bovenzi et al. (1995), Bovenzi (2008), Bovenzi et al. (2008), and Bovenzi (2010a)

For the present study, regression analyses have been performed for various data subsets for both linearly and polynomially interpolated prevalence data. The datasets considered are illustrated in Fig. 1. The full dataset is comprised of all studies deemed usable in Scholz et al. (2023a) and Scholz et al. (2023b) and is henceforth referred to as the full dataset. Those studies in which the daily usage time was observed or measured and for which the $T-ratio$ therefore equals 1, form the basis for the first subset. By including those studies in which chain saws or chain saws with vibration-isolated handles (AV-chain saws) were used, and for which the $T-ratio$ equals 1.1, the data subset $T-ratio$ 11 is formed. The data subset that was deemed most reliable in Scholz et al. (2023a) and Scholz et al. (2023b), as the studies reported the lifetime exposure $D_y$ in years and the *A*(8)-value as required by the selection rules, is called $D_y$ *A*(8) rep. in what follows. Additionally, studies possessing the information required by the selection rules and with a $T-ratio$ no greater than 1.1, are analyzed. These form a subset of the $T-ratio$ 11 and $D_y$ *A*(8) rep. subsets: This data subset is called intersec in what follows.

The full dataset allows for comparison to the models in Scholz et al. (2023a) and Scholz et al. (2023b) and therefore shows the effect of accounting for the different methods used to determine the daily usage time.

### Models

**Fig. 2:**
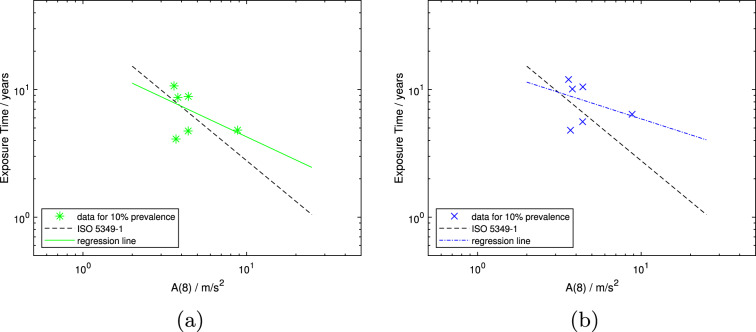
Mean population exposure times in years versus *A*(8). Data subset intersec including those studies with both a $T-ratio$ equal to 1 or 1.1, hence a basically unadjusted *A*(8), and that have also been deemed most reliable in previous analyses (Scholz et al. (2023a, 2023b)) with linearly (**a**) and polynomially (**b**) interpolated exposure times to 10% prevalence of VWF, $D_{y,10,lin}$ and $D_{y,10,poly}$ (asterisks and crosses), respectively. Both Figures show the corresponding regression line (solid green line in (**a**) and dash-dotted blue line in (**b**)) and the model from ISO 5349-1 (dashed line)

The estimated mean exposure times to reach 10% prevalence of VWF in each population, $D_{y,10, j, k}$, from Table 1 and the corresponding adjusted and unadjusted (i.e., "original") $A(8)_k$-values from Table 3 are then used to create exposure-response models using a regression analysis (Eq. 5). The results of these analyses are shown in numerical form in Table 4 and in Fig. 2 through 6.

Table 4 contains a number with which each model can be referenced, the name of the dataset or subset, the method of interpolation to 10% prevalence of VWF, the values of the fit parameters *a* and *b*, the resulting $r^2$-value and whether the original or the adjusted *A*(8)-value is used. The Figure number(s) in which each model is presented graphically is/are also listed together with the line type and color. The footnotes to Table 4 list which studies are included in which dataset.

**Table 4:** Dataset, prevalence interpolation method (linear or polynomial), fit parameters *a* and *b*, and $r^2$ for all exposure-response models predicting population exposure times in years to reach 10% prevalence of VWF identified by number, whether *A*(8) was adjusted or not, in which Figure the respective model is included and the line type and color used

No$^\circ$	Dataset or subset	Inter-polation	Fit parameter *a*	Fit parameter *b*	$r^2$	*A*(8)	Figure	Line type (color)
1	$T-ratio$ 11^a^	Lin.	23.3	− 0.86	0.64	adj.	3a	s (green)
2	$T-ratio$ 11	Poly.	22.3	− 0.70	0.50	adj.	3b	d-d (blue)
3^b^	Full dataset^c^	Lin.	20.6	− 0.74	0.69	orig.	4a, 6b	s (green)
4^d^	Full dataset	Poly.	19.4	− 0.56	0.56	orig.	4b,6b	d-d (blue)
5	Full dataset	Lin.	20.3	− 0.76	0.67	adj.	4a, 6	s (orange)
6	Full dataset	Poly.	19.6	− 0.61	0.55	adj.	3, 4b, 5, 6	d-d (magenta)

The line types are encoded (s = solid, d-d = dash-dotted)

^a^ $T-ratio$ 11:Bovenzi et al. (1995), Bovenzi (1998b), Bovenzi (2008), Bovenzi et al. (2008), Bovenzi (2010a)

^b^ From Scholz et al. (2023a)

^c^ Full dataset: all studies listed in Table 1

^d^ From Scholz et al. (2023b)

#### Analyses of the intersec data subset

In Fig. 2a and b, studies common to the $T-ratio$ 11 and $D_y$ *A*(8) rep. data subsets (i.e., the intersec data subset), which contain the data requiring the least adjustment and hence may be considered the most reliable in all respects that the authors have considered so far, are plotted, both for linearly and polynomially interpolated prevalence. As the data points are small in number and scattered, there is a regression line, but no confidence intervals are shown. The regression lines have slope (i.e., value for *b*) of -0.60 and -0.41 for linearly and polynomially interpolated prevalence, respectively. They are shown by the solid line in Fig. 2a and the dash-dotted line in Fig. 2b, but are not considered to represent meaningful exposure-response models as $r^2$ is only 0.19 and 0.12, respectively. Hence, these results are not included in Table 4. In both Figures, the dashed line shows the ISO prediction for the development of 10% prevalence of VWF in a population.

#### Models constructed from the $T-ratio$ 11 data subset

**Fig. 3:**
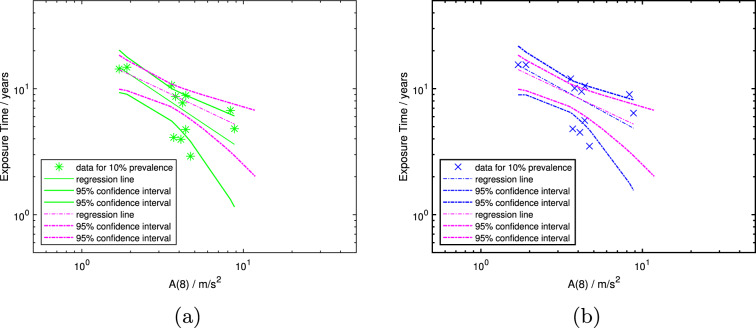
Mean population exposure times in years versus $A(8)_{adj}$. Data subset $T-ratio$ 1.1 with (**a**) linearly and (**b**) polynomially interpolated exposure times to 10% prevalence of VWF (green asterisks and blue crosses, respectively). Both figures show the corresponding regression line and 95-percentile confidence intervals of the models for this data subset (solid green lines in (**a**) for model 1, and dash-dotted blue lines in (**b**) for model 2), and for the entire dataset with polynomial prevalence interpolation (dash-dotted magenta lines) from Fig. 4b (model 6)

The $T-ratio$ 11 data subset is plotted together with the models derived from it in Fig. 3a and b. As in the previous figures, Fig. 3a shows the linearly interpolated and Fig. 3b the polynomially interpolated prevalence dataset. The exposure-response relation is shown by the solid green line in Fig. 3a for linear prevalence interpolation (model 1) and by the dash-dotted blue line in Fig. 3b for polynomial prevalence interpolation (model 2). The 95-percentile confidence intervals are shown by the thick lines of the same color in each graph. They show both differences created by the interpolation methods and how close model 6 using polynomial prevalence interpolation for the full dataset (shown by the dash-dotted magenta lines) is to models 1 and 2. Comparing models 1 and 6, the latter has more narrow confidence intervals and the regression line and the confidence intervals are tilted with respect to the former: This results in longer predicted mean exposure durations to reach 10% prevalence in a population for $A(8)_{adj}$-values greater than 2$\frac{m}{s^2}$. The regression lines of models 2 and 6 both of which use polynomial prevalence interpolation mostly overlap, while model 6 again has the narrower confidence intervals.

#### Models constructed from the full dataset

**Fig. 4:**
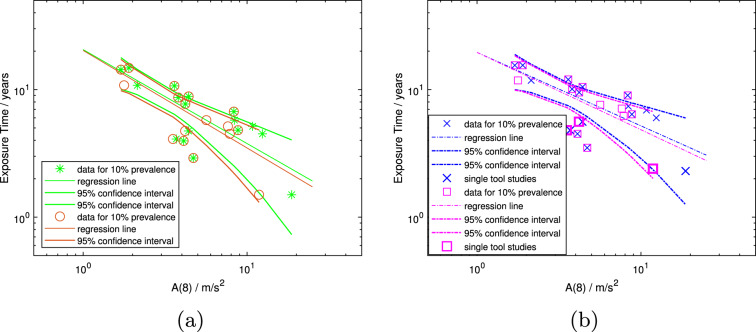
Mean population exposure times in years versus *A*(8) unadjusted and adjusted. Full dataset interpolated to 10% prevalence, regression lines forming the exposure-response models and the corresponding 95-percentile confidence intervals.** a** shows the dataset with linearly interpolated prevalence to 10% VWF for the unadjusted *A*(8)-values (green asterisks) and the adjusted *A*(8)-values (orange circles), and the corresponding models; model 3 (solid green lines) and model 5 (solid orange lines).** b** shows the dataset with polynomially interpolated prevalence to 10% VWF for the original *A*(8)-values (blue crosses) and the adjusted *A*(8)-values (magenta squares), and the corresponding models; model 4 (dash-dotted blue lines) and model 6 (dash-dotted magenta lines)

The models constructed from the full dataset are as follows: Model 3 is from Scholz et al. (2023a) and model 4 originates from Scholz et al. (2023b). Model 5 is created from the same linearly interpolated dataset as model 3, but with the adjusted *A*(8)-values listed in Table 3. Model 6 is created from the polynomially interpolated full dataset, like model 4, again just differing in the *A*(8)-values used. These two pairs of models are plotted by method of prevalence interpolation in Fig. 4a and b. They show that only five data points shift noticeably due to the *A*(8) adjustment (i.e., compare locations of circles and asterisks in Fig. 4a, and locations of squares and crosses in Fig. 4b). Therefore, the visual distinction between the two pairs of models is minimal. For models 3 and 5, the fit parameter *a* is very similar, and *b* differs by 0.02, as does the $r^2$-value. For the models that are based on polynomially interpolated data, the difference is slightly bigger for the fit parameter *b* (0.05).

### Comparison and evaluation of models

**Fig. 5:**
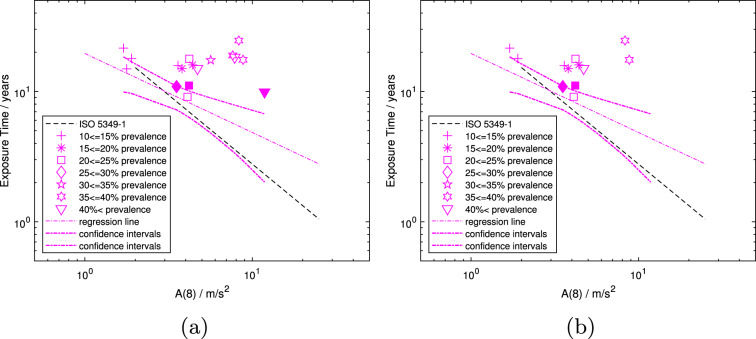
Mean population exposure times in years versus *A*(8) unadjusted and adjusted. Prevalence data as reported in the published studies, not interpolated, with the respective prevalence encoded in the symbol in 5% intervals (see legend). Filled symbols indicate studies in which a single tool or machine was used throughout the workday. Both figures show the model for the full dataset that was created after adjusting the *A*(8)-values and interpolating polynomially to 10% prevalence of VWF (dash-dotted magenta line, model 6), together with the corresponding 95-percentile confidence intervals (dash-dotted magenta curves) and the model predicting 10% prevalence from ISO 5349-1:2001 (dashed black line).** a** shows the prevalence data from all epidemiologic studies with *A*(8)-values adjusted for the daily usage times,** b** shows only those data points for the $T-ratio$ 11 data subset and hence mostly unadjusted *A*(8)-values

Figure 5a and b show model 6 with its confidence intervals in magenta and the ISO model as a dashed black line. Additionally, the figures include the uninterpolated data with the prevalences as reported in the original epidemiologic studies encoded in the symbols (see legends). In Fig. 5a, the full dataset is shown with the daily vibration exposure adjusted by means of the respective $T-ratio$s, i.e., $A(8)_{adj}$. Figure 5b includes only the $T-ratio$ 11 dataset and, hence, the daily vibration exposure as it was reported in almost all the respective studies. In both cases the regression line of model 6 lies beneath all data points with a reported prevalence of 10% or higher. Data points of various prevalences come close to both models, and in Fig. 5a, one study in which the prevalence was in the range of 10–15% lies below the model from the ISO standard.

**Fig. 6:**
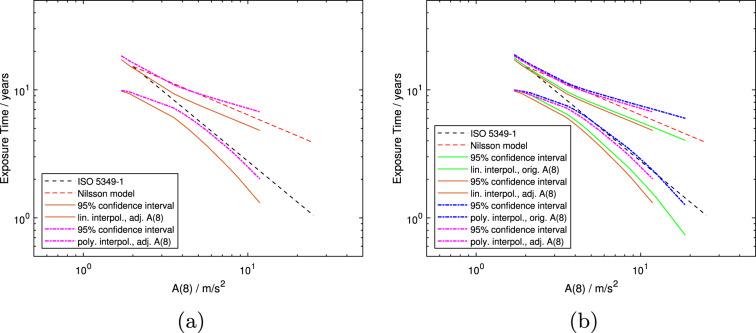
Mean population exposure times in years versus *A*(8) unadjusted and adjusted.** a** shows 95-percentile confidence intervals of models 5 and 6 created here (solid orange and dash-dotted magenta lines, respectively), as well as the model from ISO 5349-1 (dashed black line) and the model created by Nilsson et al. (2017) (dashed red line).** b** shows 95-percentile confidence intervals of models 5 and 6 created here, and of models 3 and 4 from Scholz et al. (2023a) and Scholz et al. (2023b) (solid green and dash-dotted blue lines, respectively). The green confidence intervals (solid curves) are from the model created from linearly interpolated prevalence data and unadjusted *A*(8)-values (model 3). The orange confidence intervals are from the model created from linearly interpolated prevalence data and adjusted *A*(8)-values (model 5). The blue confidence intervals are from the model created from polynomially interpolated prevalence data and unadjusted *A*(8)-values (model 4). The magenta confidence intervals are from the model created from polynomially interpolated prevalence data and adjusted *A*(8)-values (model 6)

In Fig. 6a and b, both the models from ISO 5349-1:2001 and Nilsson et al. (2017) are plotted (dashed black and red lines, respectively), as well as the 95-percentile confidence intervals of models 5 and 6 (solid orange and dash-dotted magenta lines, respectively). Figure 6b additionally shows the 95-percentile confidence intervals of models 3 and 4 (solid green and dash-dotted blue lines, respectively). The area between the upper and lower 95-percentile confidence intervals can be considered to define a region within which it is most likely that the true exposure-response relation is to be found for 10% of a population to develop VWF. In this comparison with other published work, it can be seen that the model from ISO 5349-1:2001 lies between the upper and lower confidence intervals of all these models for almost all values of *A*(8) or $A(8)_{adj}$ plotted. In contrast, the model from Nilsson et al. (2017) lies just on the upper limit of the 95-percentile confidence intervals for models created from polynomially interpolated prevalence data at low *A*(8)-values (models 4 and 6 - the blue and magenta dash-dotted lines, respectively—see Fig. 6b). However, it remains outside the region in which the true exposure-response relation is most likely to be found for models created from linearly interpolated prevalence data (models 3 and 5 - the solid green and orange lines, respectively - again, see Fig. 6b).

## Discussion

As noted in the authors’ previous pooled analyses of VWF, the number of epidemiologic studies from which comparable and reliable data can be sourced is limited. In order to allow for comparison with models from the authors’ prior work and hence to determine the influence of only the factor analyzed, the studies from which the data are sourced are kept identical to those used in the previous analyses. Thus, the epidemiologic studies judged most reliable by Nilsson et al. (2017) in their authoritative meta-analysis that also satisfy the selection rules introduced by Scholz et al. (2023a) are included here. In consequence, the limitations of the studies selected by this procedure, which are described in Scholz et al. (2023a), are accepted. These include: the small number of laboratories and countries from which the studies originated; the absence of finger blanching in vibration-exposed persons in tropical countries, which suggests that the models may not be applicable to such climates (Su et al. (2013)); and the use of the frequency-weighting proposed by ISO 5349, which is not now believed to represent the vascular hazard of hand-transmitted vibration at different frequencies (Brammer and Pitts (2012)). There also remains uncertainty in the precision of polynomial interpolation to 10% prevalence of VWF (Scholz et al. (2024)). For this reason, as in previous work, equal weight is given to linear prevalence interpolation in interpreting the results.

One additional consideration affects the *A*(8)-value, the exposure-response models, and the adjustment to the daily usage time in the present study. If the members of a population use more than one tool or machine daily, then the respective product of the daily usage time for each tool or machine and its frequency-weighted triaxial acceleration squared are combined as described by Eq. 1 to construct the overall *A*(8)-value. This implies that the uncertainties or inaccuracies in the daily usage time and the vibration for each tool are summed. As the studies used in the present work do not provide the individual values for both parameters for each tool or machine, an adjustment can only be made to the overall *A*(8)-value for a population. Without information concerning what fraction of the *A*(8)-value stems from which tool or machine, the $T-ratio$-values are given equal weight for each, and the adjusted *A*(8)-value is calculated according to Eq. 3 by means of the averaged $T-ratio$. Considering that not all tools and machines will be used for equal times during a workday, this is likely to introduce an additional inaccuracy as well. For most of the tools used in the studies for which this may be the case, the $T-ratio$ is 2.5 and therefore is assumed to have accounted for this uncertainty.

### Daily usage time adjustment

While several studies (Palmer et al. (2000); McCallig et al. (2010); Burström et al. (1998)) document the difference in daily usage time depending on the determination method (e.g., subject recall by interview or questionnaire, or from third-party observation and measurement), the influence of the adjustments on the present models appears to be limited. This may be because only roughly one third of the data points needed substantial adjustment (i.e., possessed a $T-ratio$ greater than 1.1).

Only four of the studies included in the pooled analyses did not measure or observe the daily usage time directly. In two of these, only chain saws were used and hence the adjustment is a $T-ratio$ of 1.1 (see Table 2). The insensitivity of the daily usage time to the method of measurement for chain saw operators is most likely related to the common work practice for logging: operating the tool continuously after each refill of the fuel tank until empty, together with knowledge of how long it takes for a tankful to be consumed. A third study involved rock drilling for which the maximum adjustment to the daily usage time was applied (2.5). A fourth study encompasses four populations in total, three of which used various tools, and one angle grinders (see Table 3).

There are five data points that are noticeably affected by the adjustment to the daily usage time. In all of those five cases, the *A*(8)-value was reduced through the adjustment of the daily usage time, which shifts the data points to the left in the graphs. The effect of using polynomial interpolation to estimate the mean population exposure time to reach 10% prevalence of VWF, obtained by introducing the adjusted *A*(8)-value into Eq. 4, is small (e.g., compare values for $D_{y,10,poly}$ and $D_{y,10,poly,adj}$ in Table 1).

The effects of the adjustments to *A*(8) and $D_{y,10,poly}$ can be seen in Fig. 4. In these graphs, the data points have been plotted as both unadjusted values (asterisks in Fig. 4a, and crosses in Fig. 4b), and values after adjusting for the method of determining the daily usage time (circles in Fig. 4a and squares in Fig. 4b). In both figures, the full dataset is plotted. While the shift in some data points is noticeable, the changes in the models created through the adjustments are minimal for both interpolation methods (i.e., compare green and orange lines in Fig. 4a, and blue and magenta lines in Fig. 4b).

This observation is confirmed by the results in Fig. 3. These graphs display models 1 and 2 generated by linear prevalence interpolation (Fig. 3a, green lines) and polynomial prevalence interpolation (Fig. 3b, blue lines), respectively, using the $T-ratio$ 11 dataset. Inspection of Fig. 3b reveals that using the data for which either no adjustment is necessary, or the $T-ratio$ is 1.1 and hence results in only a minimal change in the *A*(8)-value, there is an extremely small, but noticeable change in the regression lines when model 2 is compared to model 6 (i.e., compare blue and magenta lines). There is, however, a larger change in the confidence intervals. Inspection of Fig. 3a, which contains the model for the $T-ratio$ 11 data subset generated by linear prevalence interpolation (model 1, green lines) and the model for the full dataset obtained by polynomial prevalence interpolation (model 6, magenta lines) further reveals that the primary difference between the models remains the method for estimating the mean exposure time to reach 10% prevalence of VWF. Additionally, it can be seen from Fig. 3a that the 95-percentile confidence intervals are farther separated for model 1 than for model 6, indicating that the latter is more precise even though it was constructed using the full dataset, many of which studies required adjustment to the daily usage time.

Comparing the models numerically in Table 4, for all of them both fit parameters *a* and *b* are similar in magnitude. The slopes of the exposure-response relations (i.e., *b*-values) range from -0.56 to -0.86, with those for linear prevalence interpolation consistently larger than for polynomial interpolation. This is clearly evident by comparing the slopes of models 1 and 6 in Fig. 3a (i.e., green versus magenta lines). The effect of adjusting the daily usage time on the models, already discussed with respect to the graphical representation in Fig. 4, can be seen to result only in small numerical changes in slope between the original, unadjusted *A*(8) and the adjusted values for the full dataset (viz., changes in slope of 0.02 for linearly interpolated prevalence, and 0.05 for polynomially interpolated prevalence as already noted). In contrast, the differences in slope between models due to the prevalence interpolation method is approximately four times greater (ranging from 0.15 to 0.18). A similar tendency is displayed by the *a*-values. These observations indicate that the method for estimating prevalence development is more important than the method for determining daily usage times in the construction of the present models. Of course, employing a reliable method for determining the daily usage time remains critical in any epidemiologic study that endeavors to relate vibration exposure to the development of HAVS.

### Inferences from the uninterpolated data and the models

Figures 5a and b show the uninterpolated data as recorded in the original epidemiologic studies, i.e., raw data points with the prevalence encoded in the symbols. The figures show a clustering of data points with similar *A*(8)-values and mean group exposure times, but different prevalences (i.e., clustering of differently shaped symbols). Furthermore, not only do the data points within the same prevalence range not form a line, and rather are scattered over the plot, but also data points with prevalences in the range of up to 25-30% lie very close to the ISO line, which is intended to represent 10% prevalence. If the daily vibration exposure value, in this case *A*(8), were an accurate representation of the daily exposure and therefore a good measure to estimate the risk of developing VWF, then the higher the prevalence range, the further the data points would lie from a 10% prevalence exposure-response model. It would be expected that the higher the prevalence of a data point, the more likely it would be located towards the top right-hand corner of the graphs, i.e., at higher mean population exposure times and/or *A*(8)-values. Yet, in these figures, one of the three data points with a prevalence of over 40% lies between data points with prevalence ranges of 10–15%, 15–20%, and 30–35%. And data points with prevalences within the range of 30-40% are found further up and to the right.

Comparing Fig. 5a and b it becomes evident that this issue is not due to inaccuracies in the daily usage times of the tools or machines as it is present in both figures, even though Fig. 5b contains only data points with a $T-ratio$ of 1 or 1.1. Additionally, even the data from single tool or machine studies, represented by filled symbols, show a point with a range of 25–30% prevalence to the left of a data point with a range of 20–25% and hence closer to the ISO exposure-response relation for 10% prevalence of VWF. It is likely that, as stated in Scholz et al. (2023a) and Scholz et al. (2024), the single tool or machine data points will be more accurate. This is because when multiple tools are used daily the total *A*(8)-value is derived from the sum of the tools used, and hence accumulates the individual uncertainties, as already noted. However, the issue described clearly occurs with single tool or machine studies as well.

### Confidence intervals and the onset of VWF

The $r^2$-values of the models range from 0.50 to 0.69, with the values for linear prevalence interpolation consistently greater than those for polynomial prevalence interpolation for the same dataset or subset (Table 4). Counterintuitively, the model with the highest $r^2$-value is not necessarily the best exposure-response model. It only indicates how close all data points are to the regression line. The model with the highest $r^2$-value would be the best if there were no clustering of uninterpolated data points with different prevalences, as can be seen to occur in Fig. 5a and b.

A regression line, by definition, represents the "best fit" mathematically of a curve to the data points included in the model. As noted in Scholz et al. (2023b), this makes a good case for using the lower 95-percentile confidence interval rather than the regression line itself for specifying a conservative maximum exposure to hand-transmitted vibration for limiting the development of VWF. Comparing the confidence intervals of the models 3, 4, 5 and 6 in Fig. 6, the region between the upper and lower 95-percentile confidence intervals is narrower when they are all considered together. This restricted region may thus be considered to be the most likely to contain the exposure-response relation for a population to develop a 10% prevalence of VWF. An exposure limit could thus be constructed from the average of the lower 95-percentile confidence intervals.

The model from the ISO standard lies effectively within the most restricted region formed between the upper and lower confidence intervals (dashed black line), whereas the model from Nilsson et al. (2017) is mostly outside of this area (dashed red line). After adjusting the *A*(8)-values of the individual studies for their daily usage times, the uninterpolated data points in Fig. 5a are closer to the ISO line than in previous publications by the authors and one even lies below it. This last-mentioned data point has a prevalence in the range of 10–15% and previously was very close, but above the ISO model. Taking both Figs. 5a and 6 into account, it may still be concluded that the ISO model provides a conservative prediction for the onset of VWF except, perhaps, for *A*(8)-values less than about 3 $\frac{m}{s^2}$.

### Daily vibration exposure evaluation

Using only the data that are deemed most reliable, both regarding the mean group exposure time and the *A*(8)-value, as well as the method used to determine the daily usage time, reduces the dataset to six points. With such a small number of data points, a statistically meaningful model was not obtained (i.e., $r^2<< 1$). Hence, Fig. 2a and b only show the curve “fits” from the regression analysis and do not include confidence intervals. The regression lines appear to be tilted compared to those of the models in Fig. 4a and b, respectively. Both figures show data points interpolated to 10% prevalence of VWF at very similar *A*(8)-values, yet at different mean population exposure times (i.e., different ordinate values for approximately the same abscissa value). This phenomenon is most clearly seen in Fig. 2a and b, and has been observed since Scholz et al. (2023a). This points to an issue in the evaluation of the relation between vibration exposure and the development of VWF, as similar daily vibration exposures and mean group exposure times should result in a similar prevalence. Similar mean population exposure time with different daily vibration exposures, on the other hand, should result in a different prevalence. If the calculation of the daily vibration exposure value is appropriate, and the mathematical form of the exposure-response relation and the methods for determining all components are correct, then all data points should lie along the regression line of an exposure-response model. This not being the case points to issues likely within the daily vibration exposure value, which combines several possible sources of uncertainties: the daily usage time, the triaxial acceleration, and its frequency weighting, as well as how they are related to one another.

The *A*(8)-value is constructed, as shown in Eq. 1, from the daily usage time and the frequency-weighted triaxial acceleration squared. Through squaring it, a greater weight is given to the acceleration compared to the daily usage time. Griffin et al. (2003) compared different weights between the lifetime usage time (i.e., usage time per day multiplied by days per year exposed multiplied by the number of years exposed) expressed in hours and the acceleration for individual workers and found that those vibration exposure values in which the lifetime usage time in hours and the unweighted acceleration were given equal weight best described the development of VWF. Furthermore, the frequency weighting applied in forming the *A*(8)-value required by ISO 5349-1:2001 is known to be not the most suited to evaluate the vascular response of the hand-arm system (Brammer and Pitts (2012)).

And finally, the construction of the daily exposure does not distinguish between continuous and transient vibration, i.e., shocks and impacts. This liitation may also influence the magnitude of the daily exposure (Gerhardsson et al. (2020)).

## Conclusions

The exposure-response models created from the full dataset, as well as the subsets, show small, though noticeable changes due to the adjustments that account for the different methods used to determine the daily usage times. Thus, the exposure-response models created in Scholz et al. (2023a, 2023b) and in the present analyses are fairly robust to such changes. While it may be argued that this is due to only about one third of the data points being affected by the adjustment to the daily usage time, the analyses also show that the method used to determine the daily usage time is not the cause for either the clustering of uninterpolated data points with different prevalences in the graphs or for the deviation of the data points from the regression line in the plots of the interpolated data. In fact, the analyses demonstrate that the method for estimating 10% prevalence of VWF (vibration-induced white finger) in a population from the observed prevalence has more effect on the exposure-response models than the method for determining the daily usage time.

As an exposure-response model is intended to be used for worker protection, ideally, no data point should lie below it in a plot such as Fig. 5. That is, no population should experience 10% prevalence of VWF at lower lifetime exposures or *A*(8)-values than predicted by the model. The essence of a regression line is in opposition to this concept, as about half the data points will lie above and half below the line. This is why more weight should be given to the average of the lower 95-percentile confidence intervals of the models when defining a tolerable daily vibration exposure. The lower confidence intervals of the four models created from the full dataset lie either partially on, or completely below, the current model from ISO 5349-1 (2001). This indicates, together with almost all uninterpolated data points lying above it in Fig. 5, that the ISO model appears to provide a conservative estimate for an exposure limit. This conclusion was also reached previously in Scholz et al. (2023b), at least for *A*(8)-values greater than about 3$\frac{m}{s^2}$.

Nevertheless, the persistence of the observed unexplained phenomena concerning the clustering of the data points with different prevalences and the spread of data about the regression lines of the models indicate that further analyses and possibly a change in the construction of the *A*(8)-value need to be considered. This will entail analyzing both the frequency weighting applied to the triaxial acceleration and the relation between this acceleration, both continuous and transient, and the daily usage time, as well as how the *A*(8)-value is calculated for single compared to multiple tools or machines used during a workday. These issues will be addressed in future work, together with sensitivity analyses to evaluate how model input parameters influence the outcomes.

## Data Availability

Not applicable.
